# Trimethyllysine: From Carnitine Biosynthesis to Epigenetics

**DOI:** 10.3390/ijms21249451

**Published:** 2020-12-11

**Authors:** Marijn N. Maas, Jordi C. J. Hintzen, Miriam R. B. Porzberg, Jasmin Mecinović

**Affiliations:** Department of Physics, Chemistry and Pharmacy, University of Southern Denmark, Campusvej 55, 5230 Odense, Denmark; mmaas@sdu.dk (M.N.M.); hintzen@sdu.dk (J.C.J.H.); porzberg@sdu.dk (M.R.B.P.)

**Keywords:** trimethyllysine, epigenetics, post-translational modifications, protein lysine methyltransferases, protein lysine demethylases, carnitine, methylation, writers, readers, erasers

## Abstract

Trimethyllysine is an important post-translationally modified amino acid with functions in the carnitine biosynthesis and regulation of key epigenetic processes. Protein lysine methyltransferases and demethylases dynamically control protein lysine methylation, with each state of methylation changing the biophysical properties of lysine and the subsequent effect on protein function, in particular histone proteins and their central role in epigenetics. Epigenetic reader domain proteins can distinguish between different lysine methylation states and initiate downstream cellular processes upon recognition. Dysregulation of protein methylation is linked to various diseases, including cancer, inflammation, and genetic disorders. In this review, we cover biomolecular studies on the role of trimethyllysine in carnitine biosynthesis, different enzymatic reactions involved in the synthesis and removal of trimethyllysine, trimethyllysine recognition by reader proteins, and the role of trimethyllysine on the nucleosome assembly.

## 1. Introduction

**Figure 1:**
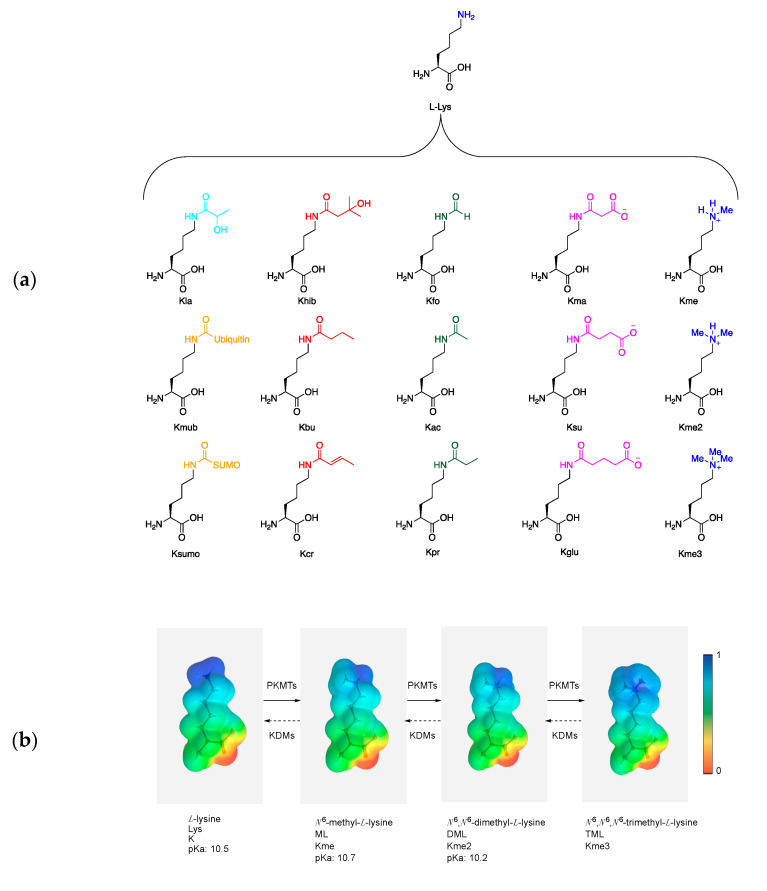
Chemical properties of L-lysine and modified lysine residues [1,8,30]. (**a**) Schematic overview of naturally occurring post-translational modifications (PTMs) of L-lysine. (**b**) Electrostatic potential surface of L-lysine and its methylated analogues. Images created with WebMO software, www.webmo.net [30].

In nature, over 300 amino acids exist, of which 22 function as building blocks for the formation of polypeptide chains called proteins [1]. Proteins are structurally and functionally diverse biomolecules that exhibit various functions, depending on the amino acid sequence and the folding into higher order structures. Proteins can act as biocatalysts, receptors, transporters, chemical messengers, and structural elements for cells. Each amino acid has a different side chain, giving each residue distinct chemical properties, such as acidity and hydrophobicity [1,2]. Humans cannot biosynthesize 9 out of the 22 amino acids [3]. One of these essential amino acids is L-lysine (Lys, K), a basic α-amino acid with a primary ɛ-amino group at the end of a 4-carbon aliphatic side chain (Figure 1a) [4]. L-Lysine is synthesized by bacteria, fungi, algae, and higher plants, from which humans obtain the amino acid through dietary means. These organisms have evolved two enzymatic pathways for L-lysine biosynthesis, namely, the diaminopimelate- and L-2-aminoadipate-mediated pathways, which have been extensively reviewed elsewhere [5,6,7].

Lysine has characteristic biochemical and physical properties. The ɛ-amino group (pKa: 10.5) possesses lone-pair electrons and exists in the protonated form at the physiological pH (7.4), giving the ɛ-amine a formal charge of +1 and a polar character that positions the residue near solvent-exposed areas of the protein, the surface, or catalytic clefts [8]. Lys can thus participate in diverse biomolecular interactions through various recognition modes including cation–π interactions, salt bridges, hydrogen bonding, and hydrophobic interactions [9,10,11]. Due to the solvent-exposed ɛ-amino group, Lys is subjected to diverse enzyme catalyzed post-translational modifications (PTMs), including methylation, acetylation, crotonylation, ubiquitination, and SUMOylation (Figure 1a). These diverse modifications can further alter its biochemical and physical properties through covalent additions, such as acetylation, inducing a loss of charge of the ɛ-amino group of lysine. Protein PTMs have the ability to fine-tune cellular processes by regulating protein stability, localization, and stimulation of protein–protein interactions [12]. Most notably, PTMs of histone proteins have been associated with an additional level of dynamic and complex regulation that controls the expression of genes in eukaryotes [13,14,15]. Crosstalk between modified Lys residues and PTMs of arginine (methylation), serine, threonine, and tyrosine (phosphorylation) regulates the epigenetic code [16].

Lys methylation is highly distinct from other histone PTMs as it is the smallest known modification and also maintains the ɛ-amine’s formal positive charge. The ɛ-amino group of lysine can be methylated up to three times—monomethyllysine (Kme), dimethyllysine (Kme2), and trimethyllysine (Kme3)—by S-adenosylmethionine (SAM)-dependent histone lysine methyltransferases (KMTs). The best studied family of KMTs is the suppressor of variegation, enhancer of zeste and trithorax (SET) domains family, which was originally characterized in *Drosophila* [17]. SET family enzymes, also classified as epigenetic “writers”, were shown to state- and site-specifically catalyze the methylation of lysine residues on histones and other proteins [18]. Non-SET domain-containing proteins, such as DOT1L, have been found to methylate Lys residues [19]. The human genome is predicted to have about 100 KMTs [20]. Each additional methylation alters the size and hydrophobicity of the side chain, which makes it possible for biological effectors to distinguish between the various methylation states.

Post-translational methylation of lysine residues in histones was previously considered to be irreversible and inheritable due to the thermodynamically stable nature of the N–CH_3_ bond [21]. Initial research revealed that the half-life of the methylation mark was similar to that of the histone protein itself, further strengthening the hypothesis that methylation leads to a persistent state of repressed heterochromatin [22]. However, discovery of various histone N^ɛ^-methyllysine demethylases (KDMs) in 2002 [23], also named “erasers”, unveiled that histone lysine methylation is a dynamic and reversible process, similar to other histone modifications, and that they play a key role in the regulation of many chromatin-based cellular processes that contribute to cellular identity and maintenance [24].

Trimethyllysine (Kme3 or TML) is characterized by a permanent positive charge due to the presence of a quaternary trimethylammonium group (Figure 1b). This broadly dispersed permanent charge makes it impossible for Kme3 to act as a hydrogen bond acceptor or donor while still being able to form salt bridges with Asp or Glu residues. The increased overall Van der Waals radius and the spatial demand necessary for cation–π interactions play a key role in specific recognition of Kme3 by biological effectors, sometimes referred as “readers”. For this reason, the trimethylammonium moiety is considered as a partially hydrophobic cloud that carries an evenly distributed formal charge of +1 [8,9,10]. Kme3-containing histones are marks for transcriptional activation and repression in eukaryotes, depending on the chromosomal context. Kme3, and other histone PTMs, act as molecular beacons for recruitment of effector/reader modules to the histones and DNA, which determine the downstream outcome of the modification and its effects on gene expression of the cell [25]. Reader motifs specific for lysine’s methylation states can recognize amino acid residues within the context of an amino acid sequence with a high degree of affinity and specificity towards their corresponding ligands. These reader domains induce selectivity towards their substrates with binding pockets that contain conserved amino acid residues with charged, hydrophilic, hydrophobic, or aromatic characteristics to attract or repulse certain PTMs [25,26]. Upon binding, various biological effects can be induced dependent on cellular context and reader protein [26].

Next to its role in proteins, Kme3 is also found in the body in its free form, and acts as a substrate in the carnitine biosynthesis pathway [27]. Carnitine is importantly involved in in the transport of long chain fatty acids from the cytosol to the mitochondria in both eukaryotes and some prokaryotes [28]. The proteolytic degradation of Kme3-containing proteins, including histones, appears to be the major source of free Kme3. Secondarily, it is also present in a variety of dietary sources [27].

Out of the various methylation states, Kme3 is of particular interest as proteins involved with the synthesis, removal, or recognition of Kme3 have been identified to contribute towards cancer development and many other human diseases [29]. Enormous effort has been made recently towards the characterization of proteins involved in the enzymatic processes generating Kme3, as well as its subsequent downstream effects on transcriptional regulation and protein function. Additionally, advances have been made towards the development of small molecule inhibitors of writers and erasers of Kme3, with some running as potential drug candidates against epigenetic diseases [29]. In this review, we aim to give an overview of the work that has been done towards elucidating the underlying biocatalytic and binding mechanisms that are involved in generation, removal, and recognition of Kme3. This review starts with an introduction to the role of Kme3 as an intermediate for the carnitine biosynthesis pathway, then it dives into catalytic processes involved in generation of Kme3, subsequent Kme3 demethylation, and Kme3 recognition. It concludes with an overview on (semi)synthetic methods to introduce Kme3 and its mimics into full-length histone proteins to study its role in the nucleosome assembly.

## 2. Carnitine Biosynthesis

**Figure 2:**
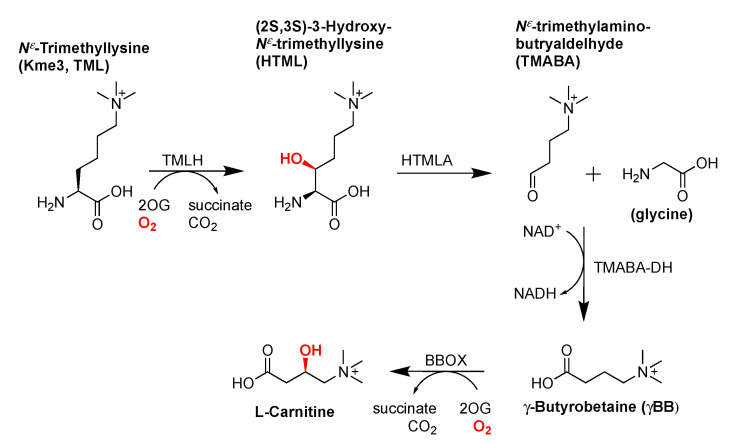
The carnitine biosynthesis pathway starting from trimethyllysine (Kme3).

Free Kme3 is involved in the carnitine biosynthesis pathway, where it acts as the first intermediate in a series of four enzymatic reactions to generate L-carnitine (Figure 2) [31]. The first step of the pathway is stereospecific C-3 hydroxylation of Kme3 to 3-hydroxy-Kme3 (HTML) by N^ε^-trimethyllysine hydroxylase (TMLH), which is the only step that takes place in the mitochondria. HTML is then transported to the cytosol and cleaved into glycine and 4-N-trimethylaminobutyraldehyde (TMABA) by HTML aldolase (HTMLA), after which it is oxidized to 4-N-trimethylaminobutyrate (γ-butyrobetaine) by TMABA dehydrogenase (TMABA-DH). In the final step, γ-butyrobetaine is hydroxylated by BBOX to the final product (3R)-3-hydroxy-4-N-trimethylaminobutyrate, better known as carnitine [31].

### 2.1. TMLH-Catalyzed C3 Hydroxylation of Kme3 and Its Role in the Carnitine Biosynthesis Pathway

TMLH is a member of the non-heme Fe(II)- and 2-oxoglutarate (2OG)-dependent oxygenases [32]. In the reaction catalyzed by these enzymes, Fe(II) acts as the cofactor and 2OG as the cosubstrate, producing CO_2_ and succinate as coproducts (Figure 2) [33]. Characteristic for 2OGXs is a fold known as the double-stranded β-helix (DSBH), which contains four major (I, III, VI, VIII) and four minor (II, IV, V, VII) β-sheets that form a squashed barrel [34,35]. The core of the DSBH can be extended by additional β-sheets, α-helices, and loops with extensive secondary structures, which are needed for substrate specificity and recognition, stabilization, and sometimes dimerization. Some extensive loops are also known to carry out catalytic activity independently [34].

**Figure 3:**
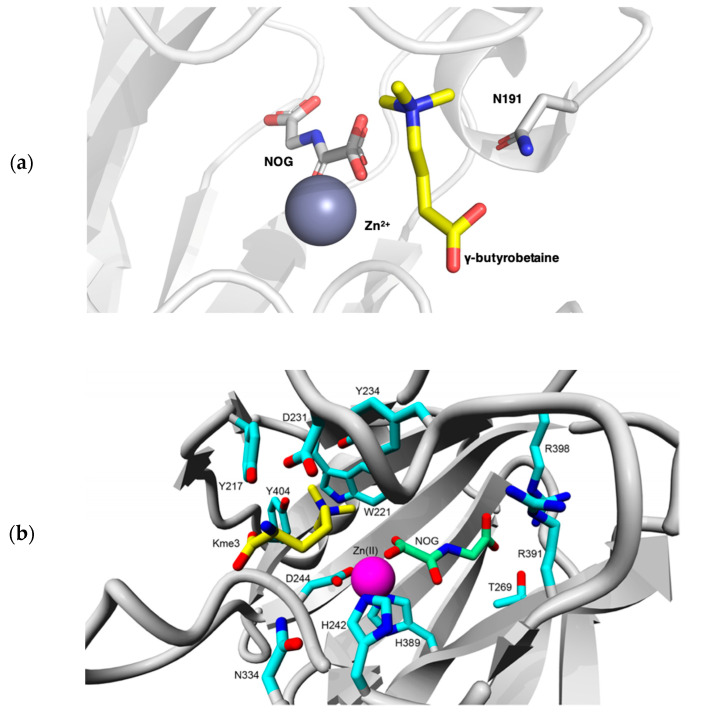
(**a**) Ribbon representations of BBOX (grey) in complex with γ-butyrobetaine (yellow) and NOG (*N*-oxalylglycine, inactive 2OG analogue). Key active site residues are labeled and zinc ions are depicted as spheres (PDB-ID: 3O2G) [36]. (**b**) The computational homology model for N^ε^-trimethyllysine hydroxylase (TMLH), based on the crystal structure of BBOX in the presence of zinc (pink), NOG (green), and Kme3 (yellow). (Reprinted (adapted) with permission from *Biochem. J.* (2019) 476 (7): 1109–1119. Copyright 2020 Portland Press.).

TMLH displays a large degree of homology with BBOX, another Fe(II)/2OG oxygenase, which catalyzes the final step in the carnitine biosynthesis pathway (Figure 2 and Figure 3a). Key residues for the catalytic activity of both enzymes include the iron chelating triad His-Asp-His, an Arg residue in the active site that forms a salt-bridge with the C-5 carboxylate of the 2OG co-substrate, and an aromatic cage consisting of Tyr and Trp residues, which is required for association with the positively charged trimethylammonium group of Kme3 (γ-butyrobetaine in the case of BBOX). Substrate specificity is thought to be achieved through the α-ammonium binding with Asp231 in TMLH, which in the case of BBOX is homologous to Asn191 [36]. As TMLH structure has not been solved yet, a computational homology model was built on the basis of the known structure of BBOX (Figure 3). This model indeed revealed that the majority of functional residues was conserved in the active site of TMLH, including the iron chelating triad, the residues responsible for binding 2OG, and the aromatic residues present in the active site. A notable difference between BBOX and TMLH is the negatively charged Asp231 of TMLH, which is located close to the α-ammonium cation of the Kme3 (Figure 3b) [37].

Early studies of the carnitine biosynthesis pathway utilized cellular lysates and basic NMR techniques to reveal Kme3 as the natural substrate, HTML as the product, and dependency on Fe(II), and 2OG and ascorbate for this reaction [38,39,40]. It was until much later that TMLH was characterized as the active enzyme from rat kidney [41]. Through using the rat sequence, homologous enzymes were found in human and mouse and confirmed to also have the TMLH activity. Rate constants of substrate and cofactor binding were determined, and it was revealed that TMLH occurs naturally as a homodimer. An active recombinant TMLH was expressed successfully, allowing for more in-depth studies of its exact catalytic activity [42].

To assess which amino acids in TMLH are crucial for binding of the Fe(II) cofactor, the 2OG cosubstrate, and the (2S)-N^ε^-trimethyllysine substrate, researchers performed site-directed mutagenesis to generate 19 variants of TMLH. It was demonstrated that several sites of recognition are important for enzymatic activity, specifically the H242-D244-H389 residues for Fe(II) chelation, the residues R391 and R398 that are involved in 2OG binding, and the aromatic cage (W221, Y217, and Y234), as well as D231 and N334 for association of the trimethyllysine substrate [37].

The (2S,3S) stereochemistry of the TMLH-catalyzed product was established through both synthetic and NMR-based investigations (Figure 2) [32,43]. The synthetic (2S,3S)-3-hydroxylysine and (2S,3R)-3-hydroxylysine were used as standards for comparison to the product formed in the presence of the recombinant TMLH. While addition of the synthetic (2S,3R)-stereoisomer to the reaction mixture revealed non-redundant peaks in 1D and 2D NMR, doping experiments in the presence of the synthetic (2S,3S)-stereoisomer showed increase in product signals, indicating that the catalytic product is the (2S,3S)-stereoisomer [32].

### 2.2. Hydroxylation of Trimethyllysine Analogues by TMLH

**Figure 4:**
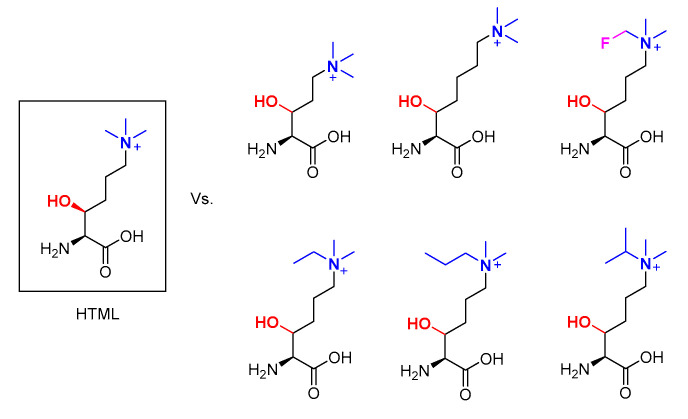
Kme3 analogues produced by trimethyllysine hydroxylase (TMLH).

In addition to mechanistic work, biocatalytic potential of TMLH was also investigated [44]. To this end, a panel of trimethyllysine analogues was examined against the recombinantly produced human TMLH. It was found that Kme3 analogues with longer or shorter side chains underwent C3-hydroxylation in the presence of TMLH. Furthermore, one of the methyl groups could be substituted by an ethyl, propyl, or isopropyl group without loss of activity. Other changes to the trimethyllysine structure were not allowed (Figure 4) [44]. Another trimethyllysine analogue that is of particular interest is the one that carries a fluoromethyl in place of one of the terminal methyl groups of trimethyllysine (Figure 4) [45]. Owing to the particular sensitivity of the 19F nucleus in NMR experiments, the fluoromethyl analogue of Kme3 was used as a probe for TMLH catalysis. It was first established that the fluoromethyl analogue indeed was hydroxylated in the presence of recombinant TMLH, as it was previously found the fluoromethylated analogue of γ-butyrobetaine was also accepted as a substrate for BBOX [46]. In the case of TMLH, the fluoromethyl analogue is accepted in cell-based assays as well as by recombinant TMLH in in vitro assays and cell lysates. It was rationalized that the three-site motif (comprised of NH_3_^+^, COO^−^, and N^+^(CH_3_)_3_) in the recognition of N^ε^-trimethyllysine is not as much perturbed by introduction of the fluoromethyl as for the two-site motif in the case of γ-butyrobetaine and BBOX [45,47].

## 3. Writing Kme3

**Figure 5:**
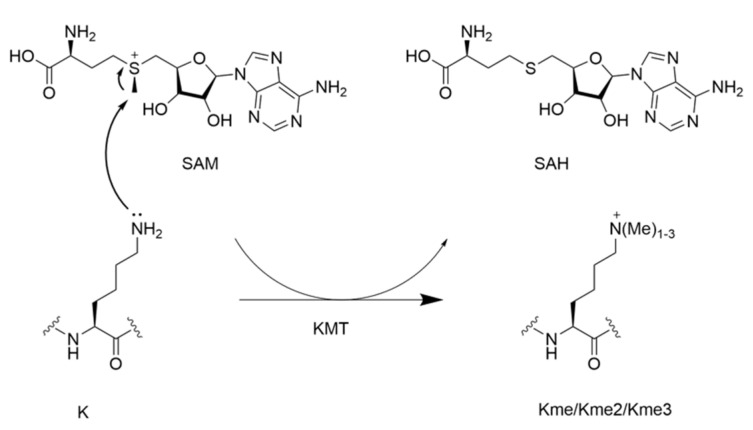
Methyltransferase (KMT)-catalyzed methylation of lysine residues in the presence of the S-adenosylmethionine (SAM) cosubstrate.

In proteins, lysine methylation is catalyzed by protein lysine methyltransferases (KMTs). An overview of proteins containing methylated lysine residues has been published elsewhere [48]. A prominent example of lysine methylation is histone methylation, which is involved in transcriptional control. Lysine methylation is only one of many PTM modifications and is a marker for various types of cancers [21]. Enzymatic methylation requires the S-adenosylmethionine (SAM) cosubstrate, which carries an electrophilic methyl group attached to a positively charged sulfur atom, facilitating the nucleophilic attack by the ε-amino group of lysine. The methylation reaction takes place via an S_N_2 mechanism, resulting in a conversion of SAM into S-adenosylhomocysteine (SAH) (Figure 5). SAM binds to the methyltransferase first, thereby forming a KMT–SAM complex that subsequently binds the substrate [49]. For each methylation state of lysine, a new molecule of SAM binds to the methyltransferase, enabling the formation of Kme, Kme2, and Kme3. The S_N_2 transition state has been extensively studied for methyltransferases SETD8 and NSD2 complex, which takes place after deprotonation, being the rate-limiting step [49,50]. In both methyltransferases, the transition state is an asymmetrical S_N_2 complex characterized by bond separation from the leaving group being at a longer distance than bond formation to the attacking nucleophile, which was found to be 2.35–2.40 Å and 2.00–2.05 Å in SETD8 and 2.53 Å and 2.10 Å in NSD2. Rubisco large subunit methyltransferase (LSMT) and SETD7 on the other hand adapt a symmetric transition state with equal distances of bond separation and bond formation. However, in all methyltransferase transition states studied thus far, methyl group positions vary, while the distance between the leaving group and nucleophile is constant [49,50].

The SET domain, a domain of approximately 130 amino acids, is responsible for methyltransferase catalysis and is found in many eukaryotic and certain bacterial proteins, and is also present in the non-histone Rubisco MTase [51,52]. The SET domain is a beta fold with curved beta strands that form small sheets. Thereby, a knot-like structure assembles next to the individual SAM and substrate-binding pockets, which together form the active site containing a cluster of aromatic residues. Several plant proteins containing Kme3 have been characterized, with Rubisco being the most prominent example [53]. In this abundant enzyme, which plays the central role in photosynthesis, K14 gets trimethylated by Rubisco large subunit methyltransferase (RLSMT), thereby regulating Rubisco’s function [54]. RLSMT is located in chloroplasts and contains, like most eukaryotic lysine methyltransferases, a conserved SET domain. The structure of Rubisco in complex with RLSMT has been studied extensively, thereby revealing a large surface-binding area between the two proteins supported by hydrophobic interactions. The Rubisco complex consists of eight small and large subunits, which together provide eight binding sites for RLSMT without sterical hindrance. At a large excess, eight molecules of RLSMT can bind to Rubisco and methylate K14 at the same time.

**Figure 6:**
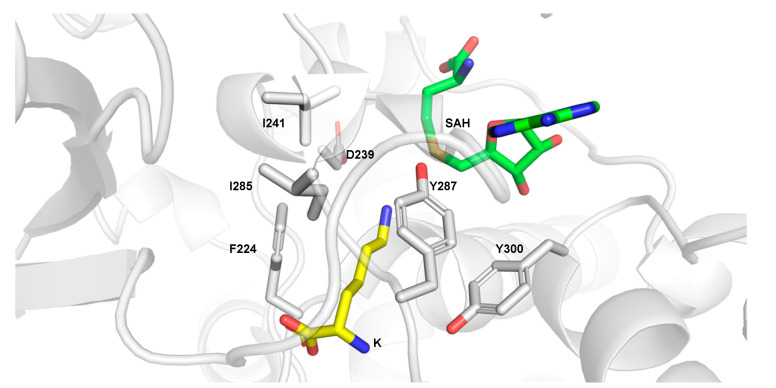
Ribbon representation of Rubisco large subunit methyltransferase (RLSMT) (grey) bound to K (yellow) and S-adenosylhomocysteine (SAH) (green). Asp239 and Ile241 stabilize binding to the substrate via a water-mediated hydrogen bond (not depicted). (PDB-ID: 1OZV (K)).

Methylation takes place upon a conformational change in a hybrid ping-pong-like mechanism, in which the methylation intermediate stays bound to RLSMT, while SAM and SAH bind and release, respectively, multiple times [51,52]. Both K and Kme are substrates of LSMT, even though their k_cat_s are significantly lower compared to the natural substrate Rubisco, while Kme2 is neither a substrate nor an inhibitor of LSMT [54]. The crystal structures of RLSMT bound to SAH and K (Figure 6) or Kme reveal that the aliphatic K side chain interacts with Phe224, Ile285, Tyr287, and Tyr300 by hydrophobic interactions. Furthermore, binding of K takes place via a hydrogen bonding with a water molecule that is stabilized by hydrogen bonding with Asp239 and Ile241. The crystal structures reveal that Kme is shifted about 1 Å compared to K, which might be due to the bulky methyl group attached. As a result, water-mediated hydrogen bonds with Asp239 and Ile241 cannot be formed, but instead a new hydrogen bond between the terminal amine of Kme and an arginine residue is formed. Interestingly, the methyl group is not surrounded by any hydrophobic residues, but instead is coordinated by carbon–oxygen hydrogen bonds in a cage containing hydroxyl and carbonyl functionalities in close proximity. In both cases, the distances between SAH and the substrates are appropriate for nucleophilic attack, as well as the angles between SAH and K and SAH (157°) and Kme (166°). These angles are optimal for an S_N_2 attack, but result in different k_cat_ of 6.2 × 10^−5^ s^−1^ for K and 2.5 × 10^−4^ s^−1^ for Kme [54].

**Figure 7:**
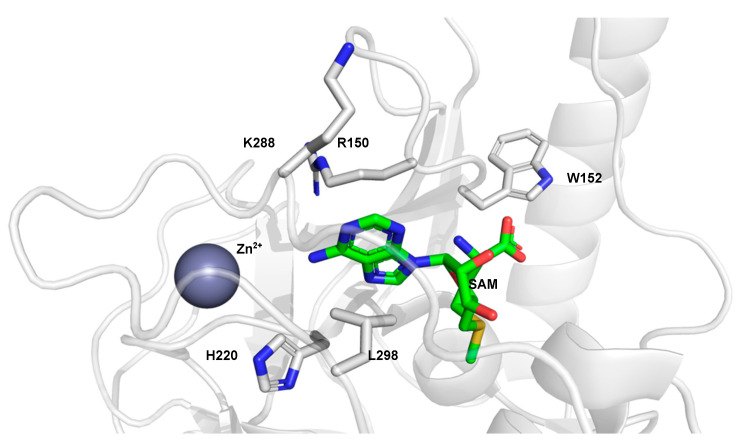
Ribbon representation of SUV39H2 (grey) in complex with SAM (green). Key binding site residues are labeled, and zinc ion is depicted as a sphere. SAM is stabilized via hydrogen bonding with K288, H220, and W152. R150 and L298 are involved in cation–π and hydrophobic interactions, respectively. (PDB-ID: 2R3A).

Histone lysine methyltransferases are mostly specific towards one methylation state. Trimethylation of K is catalyzed by the SET domain-containing proteins, the SUV39 family, the MLL family, and the non-SET methyltransferase DOT1L [55,56,57]. SUV39H2 catalyzes di- and trimethylation of H3K9, and it is structurally very similar to G9a and GLP, which catalyze mono-, di-, and trimethylation on the same K [58]. Binding of SAM by SUV39H2 is stabilized by several hydrogen bonds with amino acids located in close proximity to SAM in the binding groove (Figure 7). Furthermore, cation–π interactions with Arg150 and hydrophobic interactions with L298 contribute to the binding stability of SAM.

The peptide-binding groove of SUV39H2 is electronegative, which suggests that attraction of the positively charged histone peptide towards the enzyme takes place via non-specific, long-range electrostatic attractions [56]. Binding of the H3K9 peptide is stabilized by various hydrogen bonds with the enzyme, while the K side chain inserts in a binding channel assembled by I-SET and post-SET domains of SUV39H2 [56]. Important for enzyme activity are four cysteine residues located in the post-SET and SET domains, which coordinate a zinc ion [59]. The post-SET domain is highly conserved among the members of the SUV39 family, and coordination of the zinc ion is considered to be essential for methyltransferase activity, as demonstrated by ejection of structural zinc in G9a and GLP by small molecules [60]. However, the SET domain-containing methyltransferases SETD7 and Rubisco MTase do not contain cysteine-rich post-SET domains and their active site is formed by an alpha-helix instead of a metal center [61].

The methyltransferase activity of SUV39H2 is controlled by automethylation of SUV39H2 at Lys392, which is located in the K binding channel. The autoregulatory mechanism suggests that unmodified Lys392 has a high binding affinity towards histone 3, but when Lys392 is hyper-automethylated, methylation activity is strongly reduced [59]. The same automethylation mechanism was observed in Clr4, a yeast homologue of the SUV39 family, which contains an internal loop that blocks the H3 substrate-binding pocket. Upon automethylation of Lys455 located in the loop, a conformational change is induced. As a result, an open catalytic cage is formed, which leads to an increase in enzyme activity [62]. Another member of the SUV39 family is DIM-5, a methyltransferase that catalyzes trimethylation of H3K9. Since Kme3 is the only product of DIM-5 methylation and only traces of mono- and dimethyllysine were observed, it is proposed that the target K stays bound to the binding pocket, while SAH leaves the binding pocket, thereby enabling SAM to enter [61]. The SET domain-containing MLL family consists of the methyltransferases MLL1-MLL4, SET1A, and SET1B. Not all MLL methyltransferases catalyze trimethylation, but all of them predominantly methylate H3K4 [57].

Crucial for catalytic activity is a multiprotein complex consisting of the four subunits WDR5, RbBP5, Ash2L, and Dpy-30, the so-called WRAD complex, which binds to MLL’s SET domain [63]. A similar multiprotein complex is needed for methyltransferase activity in yeast, which is called COMPASS and contains Swd1, Swd3, Bre2, Sdc1-A, and Sdc1-B [64]. Upon mutation or downregulation of either WRAD or COMPASS, catalytic activity of the respective methyltransferase strongly decreases [57]. The SET domain of MLL1 and MLL4 only possess a sequence identity of 47%, and while MLL1 is a mono-, di-, and trimethyltransferase for H3K4, MLL4 catalyzes monomethylation more efficiently than di- and trimethylation. Their distinct substrate preferences arise from the crucial I-Y-M-F motif in the SET-I region, which, in the case of MLL4, is located 3.3 Å closer to the conserved active-site Tyr5512 than in MLL1. Consequently, the substrate lysine side chain is limited in its movement, thereby increasing the efficiency of the monomethyl transfer reaction. However, upon incubation with WRAD and unmodified H3, H3K4 is fully trimethylated, yielding H3K4me3 as the only product. In the presence of the multiprotein complex WRAD, MLL1 and MLL4 show almost identical catalytic activity in a H3 peptide assay (K_M_ 100 µM and k_cat_ 2.35 min^−1^ and 2.65 min^−1^), whereas their activity is significantly weaker without WRAD [57].

DOT1L is a trimethyltransferase for H3K79 that, unlike most other lysine methyltransferases, does not contain a catalytic SET domain [19,55]. Methyltransferase activity of DOT1L is regulated by ubiquitination of H2BK120. DOT1L binds to both ubiquitinated and unmodified nucleosomes via multiple anchors on the nucleosome surface. However, when bound to the ubiquitinated nucleosome, catalytic efficiency of H3K79 methylation is increased. During methylation, a conformational change occurs, with DOT1L staying bound to the nucleosome [65]. The SAM-binding pocket of DOT1L, which assembles into narrow channel, has a high sequence similarity with protein arginine methyltransferases. The positively charged methyl group of SAM interacts with negatively charged residues on the inside of the binding pocket, while the adenine moiety inserts in a region containing hydrophobic residues. Binding of SAM is facilitated by several hydrophobic and Van der Waals interactions, as well as hydrogen bonds. The lysine-binding channel, which points towards the SAM-binding pocket, generates hydrogen bonds between multiple conserved tyrosine, glutamic acid, and glutamine residues. Crucial for methyltransferase activity are the residues Thr139, Asn241, Ser269, and Tyr312, which form a channel that is large enough to make room for mono-, di-, and trimethyllysine. The overall negative environment facilitates deprotonation of K. During multiple methylation reactions towards trimethyllysine, H3K79 is able to stay bound to DOT1L, while SAH is released and SAM enters the binding pocket, since both binding channels operate independent of each other [19].

Notably, SET domain-containing methyltransfearses only share two conserved tyrosine residues in the active site, while additional residues determine their substrate specificity. These structural differences have been used to transform monomethyltransferase SETD7 into a trimethyltransferase by mutating active site residues. Tyr245 plays a key role in SETD7 methyltransferase activity by undergoing hydrogen bonds with lysine, thereby stabilizing binding of the substrate, and at the same time positioning Kme in a favorable orientation that sterically excludes Kme2 and Kme3 [66]. However, Tyr245 in SETD7 is substituted by proline and valine in dimethyltransferase G9a and trimethyltransferas SUV39H1, respectively, which creates additional space for the binding of Kme and even Kme2. Mutational studies that substituted Tyr245 by Ala converted SETD7 into a methyltransferase that catalyzes methylation of both Kme and Kme2, while enzymatic activity towards K decreased [66]. The same principle has been applied to trimethyltransferase DIM-5, which, upon mutation of Phe281 into Tyr, was converted into a mono- and dimethyltransferase without losing its overall catalytic activity [61]. Mutational studies on G9a, SETD8, MLL, and EZH2 confirmed that active site Tyr and Phe residues control methyltransferase product specificity [67].

### 3.1. KMT-Catalyzed Formation of Kme3 Analogues

**Figure 8:**
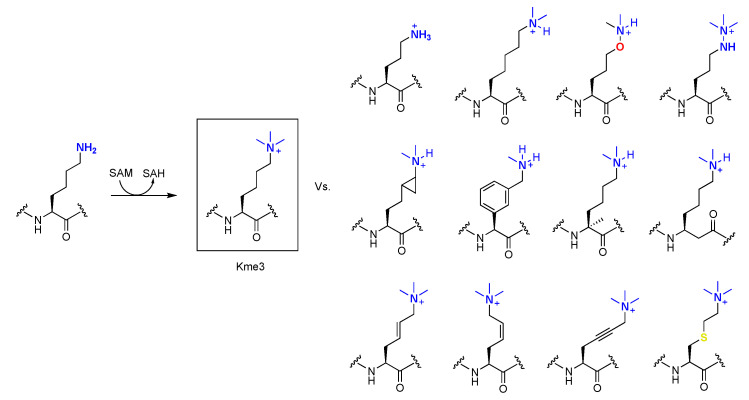
Kme3 analogues produced by histone lysine methyltransferases G9a and GLP.

To obtain a better understanding of the enzymes that catalyze the methylation of lysine residues in proteins, research effort has been put towards incorporation of lysine analogues into peptides and proteins. Such results reveal which properties of the substrate are important for the catalysis and provide deeper knowledge about the way they function. Many of these efforts have focused on the epigenetically relevant histone proteins, but one might speculate these results can be extended to non-histone substrates. As for the epigenetic writing process, trimethyltransferases G9a and GLP have been used as the model enzymes for examinations of conversions of H3K9 to H3K9me3 [17]. Setting out to elucidate the specific mechanisms of G9a/GLP, various lysine analogues have been investigated as potential substrates. D-lysine, in which the stereochemistry of the side chain is reversed, was tested against selected KMT enzymes. It was shown that the tested methyltransferases are specific for the L-enantiomer over the D-enantiomer, and that the peptide containing D-lysine is only very poorly methylated [68]. The chain length of the lysine side chain was varied as well and challenged against these enzymes in the same way (Figure 8) [69]. It was found that incorporating the one carbon shorter analogue (ornithine, Orn) led to no observed methylation for both enzymes, whereas incorporating homolysine (hLys) led to detectable amounts of monomethylation for both G9a and GLP, and dimethylation for G9a. Competition assays between the natural peptide and the Orn- or hLys-containing peptide caused significant decrease in methylated product formation on the natural peptide. In the case of Orn, decreased Lys methylation was observed in the competition study. For hLys, predominantly dimethylated lysine was formed with minor monomethylated observed. These results indicate that these peptides act as competitive inhibitors for G9a and GLP. The nucleophilic amino group was another property of lysine that was evaluated [70]. A panel of lysine analogues was used, replacing the ε-amino group by other nucleophilic functionalities (Figure 8). Of these, it was found that the aza variant was trimethylated with detectable amounts of dimethylation present, whereas the oxyamine showed only dimethylation for both G9a and GLP. Interestingly, the hydroxyl variant showed no detectable methylation. These results indicate the nucleophilic character and the basicity of lysine, as well as the conformations of the substrates, are important determinants for substrate specificity in these enzymes.

The next property of lysine that was studied is the effect of introducing more sterically demanding analogues into histone peptides [71]. These included a panel of six sterically demanding lysine analogues, ranging from small modifications as in cyclopropyllysine, introduction of a benzene ring into the side chain of lysine in various positions, an aniline derivative with a less nucleophilic terminal amino group, and tyrosine with a potential to undergo O- and C-methylation. From these results, it appears that only minor introduction of steric hindrance into the catalytic pocket of KMTs is allowed, as the cyclopropyllysine was catalyzed into the monomethyl state by SETD8 and predominately into the dimethyl state by GLP and G9a. Of the other analogues, only the highly nucleophilic benzylamine was converted into the monomethyl species, whereas for the other analogues, no methylation was observed.

Further evaluating the requirements for effective transfer of the methyl group to unnatural lysine analogues, researchers also modified the main chain of lysine and challenged it against G9a and GLP (Figure 8) [72]. It was found that the analogue carrying a methyl group on the C_α_ predominantly underwent dimethylation and to some extent mono- and trimethylation in the presence of both enzymes. However, when the methyl group was moved to the N_α_ position, catalytic activity was completely abolished for both enzymes, suggesting H-bonding between the lysine’s main chain NH and the enzymes’ main chain CO is crucial for catalytic activity. β-Homolysine was then used to further establish the importance of this interaction; however, it was found that the enzymes were still capable of generating the dimethylated state as the major product, with trace amounts of mono- and trimethylated products observed.

Finally, more rigid analogues of lysine were introduced into the same histone peptide in order to assess if the restriction of movement inside of the catalytic pocket would affect the ability of KMTs to methylate lysine [73]. The geometrically constrained lysine analogues that were introduced have an unsaturated bond between the γ- and δ-positions of lysine’s aliphatic chain. Both the (*E*)-configured and (*Z*)-configured double bonds as well as a triple bond were synthesized and introduced into histone peptides. This study revealed that the (*E*)-configuration is the preferred geometric orientation for KMTs, as this analogue was well methylated by all three studied enzymes, being catalyzed to its full extent. On the contrary, the (*Z*)-isomer was catalyzed predominantly to the dimethylated state by G9a and GLP, and not accepted by SETD8 at all. The analogue containing a triple bond, resulting in a straight orientation between the γ- and δ-positions, was not methylated by SETD8 either, but produced a mixture of the dimethylated and trimethylated states by G9a/GLP.

A particularly interesting analogue of lysine is γ-thialysine. This residue can be alkylated from cysteine to generate numerous modified amino acid residues carrying groups that can mimic post-translational modification [74,75]. As there are only one or two cysteines present in all histone proteins, cysteine alkylation can be used as a site-specific and straightforward tool to incorporate mimics of PTMs. However, there are slight differences between the natural lysine and γ-thialysine, and thus it has to be evaluated if γ-thialysine is indeed a good mimic for methylation by histone lysine transferases. The ε-amine of thialysine is a slightly stronger base (ΔpKa 1.1), and the side chain is slightly longer (0.3 Å) and angled (C−S−C angle 99° vs. C−C−C angle 109°), resulting in more degrees of freedom and functional consequences [76]. To this end, histone peptides were again challenged against G9a and GLP and it was found that natural lysine and unnatural γ-thialysine exhibit similar catalytic efficiencies owing to the fact that γ-thialysine can be used for future studies [76].

### 3.2. KMT-Catalyzed Formation of Kme3 Mimics in the Presence of SAM Analogues

**Figure 9:**
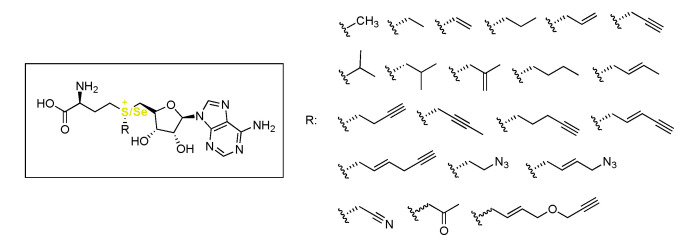
Library of SAM analogues used in biomolecular studies of SAM-dependent enzymes.

Another possibility to incorporate Kme3 mimics into proteins in the presence of KMTs is to change the cosubstrate SAM instead of the substrate peptide [77,78,79] (Figure 9). It was shown that KMTs display some promiscuity when it comes to the SAM cosubstrate structure, and through synthesizing SAM-analogues, different functional groups can be attached on lysine. An example of this is using a propargyl-functionalized SAM analogue that can be synthesized in one step from SAH [80]. This compound was used to perform CuAAC-based click reactions for labeling and profiling genome-wide methylation [81]. The repertoire of available SAM analogues was expanded by inclusion of other alkyne-functionalized groups to allow for further fine-tuning of reactivity [82]. As with the propargyl variant of SAM, the other reactive group of the classic click reaction was also successfully incorporated by KMTs, and azido-SAM could be used as a probe for genome-wide profiling of methylation as well [83].

To further expand the scope of possible SAM (also known as AdoMet) analogues that can be introduced, research found that replacing the sulfur atom of SAM by a selenium (AdoSeMet) can help increasing the effectivity of incorporation of other alkyl groups by methyltransferases [84]. AdoSeMet analogues that were incorporated include a variety of alkyne and alkene derivatives (Figure 9) [85]. As with regular SAM, propargyl-AdoSeMet could be used as a reporter of protein methylation [84,86]. Specifically, G9a and GLP were also used to study the possibility of ethylation by employing adenosylethionine (AdoEth) and adenosylselenoethionine (AdoSeEth) as the cosubstrates instead of SAM. It was found that the mono-ethylation event occurred, but further ethylation to the di- and tri-ethylated states was not found. Computational studies revealed the more optimal alignment of the smaller methyl group of SAM in the catalytic pocket of these enzymes, owing to the poorer catalytic activity using the ethylated analogues. Interestingly, G9a/GLP-catalyzed mono-ethylation of the one-carbon-shorter ornithine, and ethylation of Kme2 to Kme2et was also observed when using AdoSeEth as the cosubstrate [87].

## 4. Erasing Kme3

More than 20 different KDMs have been functionally and structurally characterized [88]. These KDMs can be divided in two families depending on their sequence homologies and distinct catalytic mechanisms: flavin adenine dinucleotide (FAD)-dependent lysine-specific demethylases (LSDs) and Fe(II)- and 2-oxoglutarate (2OG)-dependent Jumonji (JmjC) domain-containing enzymes [89,90]. On the basis of structural and biochemical investigations, research has elucidated the mechanism of action and substrate specificity of the various enzymes within the two subfamilies [24,91].

### 4.1. FAD-Dependent Lysine-Specific Demethylases Remove Methyl Group in Kme1 and Kme2

**Figure 10:**
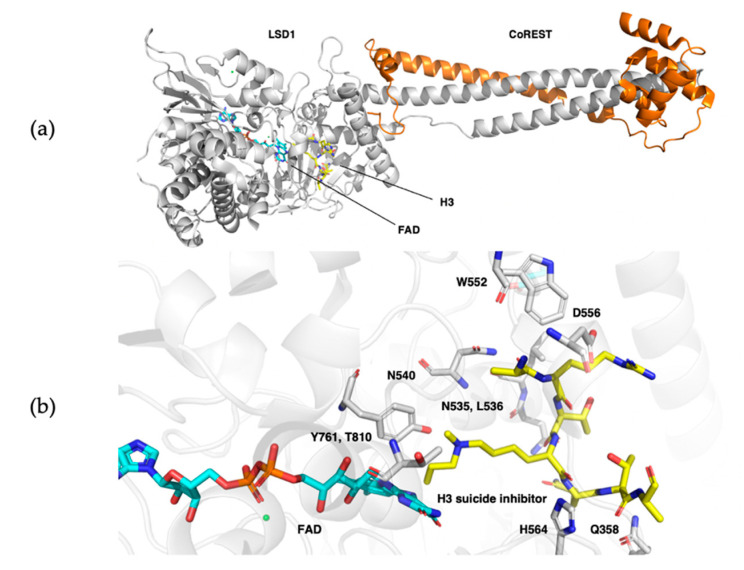
(**a**) Ribbon representation of lysine-specific demethylase 1 (LSD1)–co-repressor of RE1-silencing transcription factor (CoREST) in complex with a flavin adenine dinucleotide (FAD)-H3 suicide inhibitor (PDB-ID: 2UXN). LSD1 (grey), CoREST (orange), FAD (cyan), H3 suicide inhibitor (yellow), Cl- ions (green). (**b**) Key residues of the LSD1-H3-binding domain in complex with a FAD-H3 suicide inhibitor for LSD1 (grey). FAD (cyan), H3 suicide inhibitor (yellow) [103].

**Figure 11:**
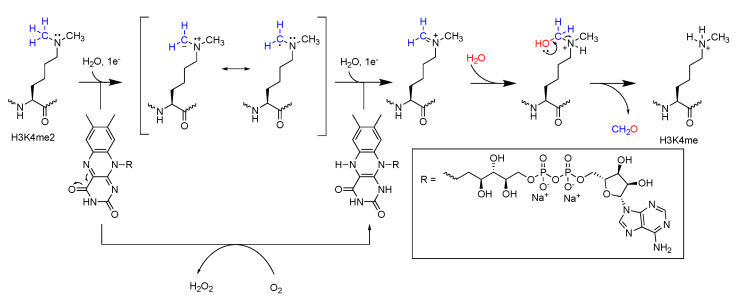
Mechanistic representation of flavin adenine dinucleotide (FAD)-dependent demethylation of dimethylated L-lysine residues by LSD1 and LSD2. LSD-catalyzed demethylation starts with flavin-mediated two-electron oxidation of methylated lysine, forming an imine intermediate upon reduction of the flavin cofactor. Subsequent hydration forms the N,O-hemiacetal, which results in collapse of the intermediate and formation of formaldehyde and demethylated substrate. FAD can be re-oxidized by molecular oxygen, releasing hydrogen peroxide from the catalytic cleft. This oxidation resets the active site back to its original state, ready to catalyze another demethylation [92].

Lysine-specific demethylases (LSD) were the first characterized demethylases of methylated histone lysine (Figure 10a). Mono- and di-methylated lysine are demethylated in a flavin adenine dinucleotide (FAD) cofactor-dependent manner [89]. LSD1 and LSD2 are characterized by their N-terminal Swi3p, Rsc8p, and Moira (SWIRM) domain as well as a C-terminal AOL (amine oxidase-like) domain, which facilitates FAD cofactor and substrate binding. These domains form a globular core through hydrophobic interactions from which, in the case of LSD1, a Tower domain protrudes with an elongated helix-turn-helix motif that plays a key role in recruitment of neuronal silencer co-repressor of RE1-silencing transcription factor (CoREST). The SWIRM domain is hypothesized to regulate protein stability and recruitment of DNA, transcriptional protein complexes, or other proteins [92]. The large catalytic center of these enzymes is formed by two lobes of the AOL domain, which enables protein–protein interaction between a longer part of the lysine-containing substrate and the catalytic cavity through hydrogen bonding and Van der Waals interactions [93]. Additionally, the size of the cavity makes it difficult for the protein to distinguish between the various methylation states of methylated lysine-containing substrates, which makes the enzyme itself non-selective towards methylation states [91,92]. Selectivity towards these methyl states is achieved through the catalyzed demethylation reaction, as it requires a free electron pair on the methylated lysine residue. Therefore, demethylation can only take place in presence of Kme or Kme2, but not Kme3 (Figure 10b). Selectivity towards substrates is achieved by the cleft between the SWIRM and AOL domains, which acts as recognition site for the terminal domains of its substrates [94,95]. However, even though LSD1 and LSD2 share mostly homologous catalytic domains, LSD2 is not able to target substrates other than H3K4me1/2 as it lacks the Tower domain. LSD1 was shown to target H3K4me1/2 and H3K9me1/2, along with non-histone targets such as P53K370me1/2, DNMT1K1096me1/2, or E2F1K185me1/2 for demethylation [96,97,98,99]. Enzymes from the LSD family catalyze demethylation of their (non-)histone substrates [91] through simultaneous amine oxidation and flavin reduction, followed by subsequent re-oxidation of flavin by one equivalent of molecular oxygen, forming stoichiometric hydrogen peroxide, formaldehyde, and the demethylated substrate (Figure 11) [100,101]. Catalysis starts with flavin-mediated two-electron oxidation of methylated lysine, forming an imine intermediate upon reduction of the flavin cofactor. Subsequent hydration forms the N,O-hemiacetal, which results in collapse of the N-hydroxymethyl intermediate and formation of formaldehyde and demethylated substrate [101]. FAD can be re-oxidized by molecular oxygen, releasing hydrogen peroxide from the catalytic cleft. This oxidation brings the active site back to its original state, ready to catalyze another demethylation reaction [102].

### 4.2. JmjC-Dependent Oxygenases Catalyse Removal of Methyl Group in Kme3

Characterization of LSD1 led to the subsequent discovery of a family of lysine demethylases that were capable of demethylating Kme3 residues [104]. The Jumonji C domain-containing demethylases (JmjCs) are members of the Fe(II) and 2-oxoglutarate-dependent (2OGs) dioxygenase superfamily, which catalyze hydroxylation, dealkylation, desaturation, epoxidation, epimerization, cyclization, and halogenation of diverse macromolecular substrates (proteins, nucleic acids, and lipids) and small molecules [33]. Most commonly, JmjCs mediate demethylation of N- or O-methylated biomolecules through hydroxylation [33]. This superfamily of KDMs is involved in epigenetic processes, and mutated enzymes have been indicated to play key roles in progression of genetic and mental disorders, midline defects, and cancer [105]. The superfamily can be divided into six subfamilies (KDM2/7, KDM3, KDM4, KDM5, and KDM6), which vary in sequence and selectivity towards nucleosomal substrates [33]. A subset of family members was observed to have N-methyl-arginine demethylation activity [106]. However, no demethylases have been found that specifically demethylate methylated arginine residues, although early work suggested that JMJD6 acts as ariginine demethylase [107,108].

**Figure 12:**
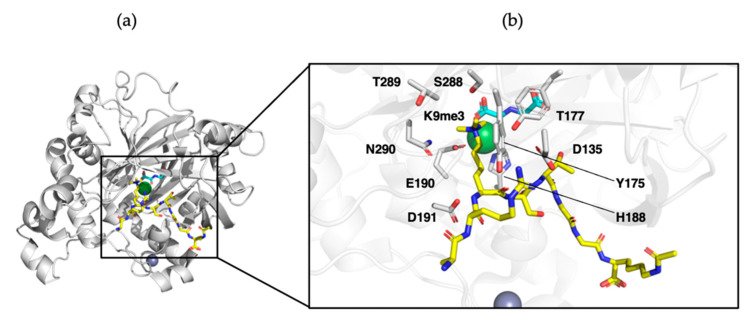
(**a**) Ribbon representation of JMJD2A (grey) in complex with H3K9me3 (yellow), Ni(II) (dark gray), Zn(II) (green), and NOG (cyan) in stick representation. (**b**) A zoomed view on the JMJD2A H3 binding site. All key JMJD2A residues are labeled [110].

Jumonji histone demethylases (JHDMs) are characterized by an eight-stranded (I-VIII) double-stranded B-helix (DSBH, or jumonji C fold). The DSBH has four major (I, III, VI, VIII) and minor (II, IV, V, VII) β-sheets, which form a squashed barrel binding element that shields cosubstrates Fe(II) and 2OG from the solution. Two histidinyl residues and a glutamyl/aspartyl residue in a conserved HxE/DxH triad allow for the coordination of Fe(II). 2OG occupies a distinct pocket, which is less conserved between family members, and the 2OG cosubstrate coordinates the iron in a bidentate manner via its 2-oxo group and one of its 1-carboxylate oxygens. The 5-carboxylate is usually bound to the side chain of a basic residue (Arg/Lys) and to a hydroxyl group from a Ser/Thr or Tyr residue [109]. Additional secondary elements surrounding the DSBH core define the various subfamilies. This includes additional β-strands, which further augment the core and inserts between β-strands IV and V. Additionally, α-helices at the N-terminus of the DSBH core augment the fold’s stability and play a role in enzyme dimerization. α-Helices located at the C-terminus determine substrate specificity and dimerization (Figure 12a).

**Figure 13:**
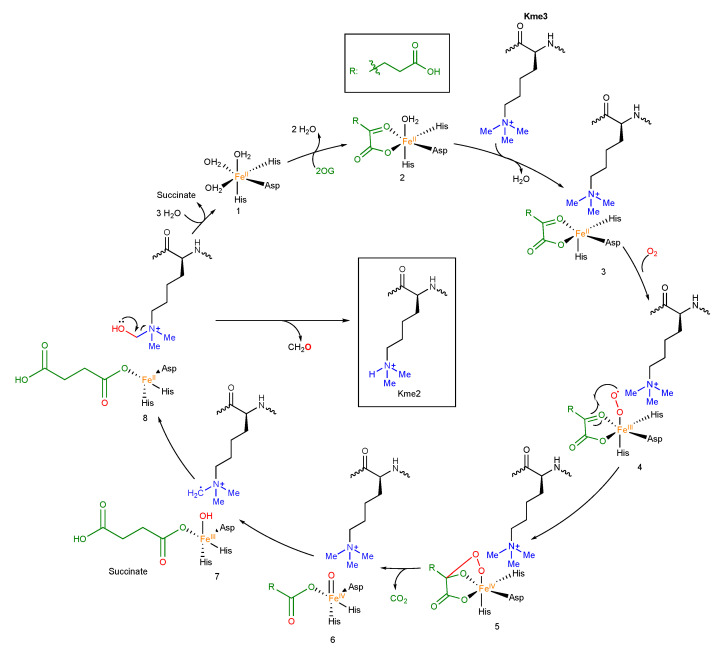
Consensus mechanism for Jumonji histone demethylase (JHDM)-catalyzed demethylation of Kme3. (**1**) Bidentate coordination of 2OG to Fe(II) is followed up by (**2**) substrate binding and (**3**) weakened Fe(II)-water coordination, opening a coordination site for O_2_ and subsequent formation of a Fe(III)-superoxide intermediate. (**4**) Oxidative decarboxylation by the distal oxide results in the formation of a bicyclic intermediate and an Fe(IV)–oxo intermediate before (**5**) CO_2_ is released from the active site. (**6**) Fe(IV)–oxo reacts with the substrate C–H to yield an Fe(III)–OH. (**7**) A radical substrate removes the hydroxyl from the Fe(III)–OH complex (**8**) before being dispelled from the active site, resetting it back to its original state.

Substrate recognition by JHDMs is often mediated by additional reader domains (function, structure, and mode of recognition discussed in a separate chapter) [111]. The catalytic consensus mechanism for the JHDMs is proposed to have an eight-step catalytic cycle (Figure 13). 2OG enters its binding pocket (**1**) and coordinates to the Fe(II) (**2**). It is suggested that formation of the Fe(II)– 2OG–substrate complex (**3**) leads to weakening of Fe(II)–water coordination, opening a coordination site for O_2_ and subsequent formation of a Fe(III)–superoxide intermediate (**4**). It is still unclear whether O_2_ binding takes place trans to either the proximal or distal histidine. The distal oxide attacks the 2-C of 2OG, which results in the formation of a bicyclic intermediate (**5**) and Fe(III), losing another electron, becoming an Fe(IV) oxo species (ferryl). Oxidative decarboxylation of 2OG results in the loss of CO_2_, giving a ferryl–oxo–succinate complex (**6**). The ferryl intermediate removes a hydrogen from the substrate’s unactivated C(sp^3^)–H bond, forming a radical substrate and Fe(III)–OH (**7**). The substrate radical subsequently removes the hydroxyl from the Fe(III)–OH complex (**8**), giving the hydroxylated product along with succinate, which dissociate and leave the active site. A slight variation of the mechanism proposes Fe(III)–OH (**7**) to be deprotonated, resulting in a Fe(II)–alkoxo intermediate. Subsequently, protonation and dissociation yield the hydroxylated substrate. Both pathways result in a full catalytic cycle, which returns the active site back to its original state. Variations on the consensus mechanism have been reported, which vary in succinate or product release from the active site, but the presented model is applicable to most JHDMs [33,34,112]. The family of JHDMs encompasses the largest family of histone KDMs. In contrast to FAD-dependent KDMs, JHDMs can demethylate all of lysine’s positively charged methylation states due to not requiring a lone electron pair on the ɛ-amino group [111]. Demethylation follows the aforementioned mechanism in which the N^ε^-methyl group is hydroxylated to yield an unstable hemiaminal intermediate, which degrades to form formaldehyde and demethylated lysine [33].

Structural analysis of KDM4A bound to its substrate H3 demonstrated the selectivity of JHDMs towards specific methylation states. Its methylammonium binding pocket contains the carbonyl oxygen of Gly170, as well as the side chains of Tyr177, Glu190, Ser288, and Asn290 (Figure 12b). Hydrogen bonding between these residues and the polarized methyl groups of trimethylated H3K9 or H3K36 directs one of the methyl groups towards the iron center, where it subsequently undergoes hydroxylation [113]. The methyl groups are directed away from the iron center when the substrate is mono- or dimethylated, resulting in slower oxidation kinetics compared to trimethyllysine. It is hypothesized that Ser288 in KDM4A dictates its specificity for trimethylated lysine, while the serine is substituted for alanine in KDM4D, stimulating specificity to dimethyl- or mono-methyl lysine [110,113,114]. Further substrate specificity towards methylated substrates by JHDMs is mediated by non-catalytic reader domains that regulate the enzyme catalysis through protein–protein or protein–nucleic acid interactions [33].

### 4.3. Demethylation of Kme3 Analogues

**Figure 14:**
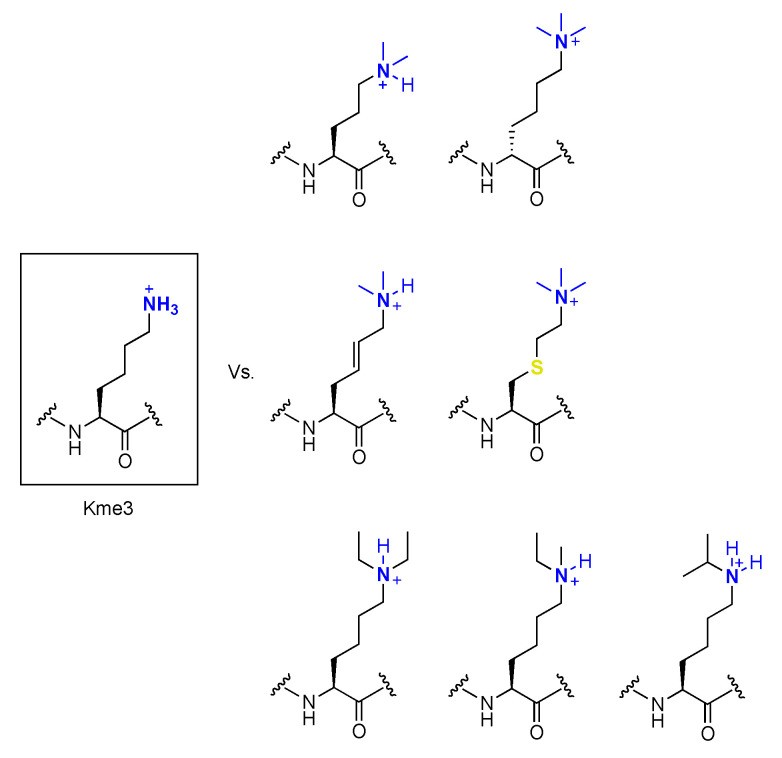
Kme3 analogues used in examinations by histone lysine demethylases.

A panel of six human histone lysine demethylases (KDM1A_JHDMs_, KDM5B_JHDMs_, KDM5C_JHDMs_, KDM4A_JHDMs_, KDM4D_JHDMs_, and KDM4E_JHDMs_) was screened for selectivity towards methylated D-lysine residues. Histone peptides carrying a trimethyllysine on H3K4 or H3K9 in either the L- or D-configuration were synthesized and incubated with KDMs. Under the tested conditions, only trace amounts of demethylation were observed, strongly suggesting that L-stereochemistry is crucial for demethylation of substrates [68]. Since other forms of Lys alkylation PMTs, such N^ɛ^-formyl-, N^ɛ^-acetyl-, or N^ɛ^-crotonyl lysine, have been observed on histones, the question arose as to whether histone lysine demethylases exhibit promiscuity towards differently alkylated Kme2/Kme3 analogues (Figure 14). To this end, a set of JHDMs was chosen to cover both H3K9 and H3K36 demethylation. The enzymes JMJD2E, PHF8, and FBXL11 were able to demethylate N^ɛ^-methyl-N^ɛ^-ethyllysine, whereas JHDMs were observed to not catalyze de-ethylation in the case of the diethyl variant [115]. Substrates containing N^ɛ^-isopropyllysine were still effectively dealkylated by the evaluated enzymes. Interestingly, N^ɛ^-isopropyllysine and natural Kme-containing peptides were observed to compete for demethylation catalyzed by JMDJ2E, suggesting promiscuity towards differentially alkylated substrates. However, JHDMs were shown to be unable to de-alkylate substrates containing N^ɛ^-formyl-, N^ɛ^-acetyl-, or N^ɛ^-crotonyl [115]. In a separate study, the histone demethylase KDM6B was evaluated with a set of histone peptides bearing several unnatural analogues [116]. It was shown that KDM6B can efficiently demethylate N^ɛ^-diethyllysine, N^ɛ^-monomethylmonoethyllysine, and N^ɛ^-isopropyllysine. Furthermore, this enzyme was shown to be able to catalyze multiple oxidation steps before the removal of the alkyl groups. It was found that KDM6B can catalyze the formation of aldehydes and even carboxylic acids in some cases.

The importance of Kme3′s aliphatic side chain conformation and charged trimethylammonium group towards JHDM-mediated catalysis of Lys demethylation was investigated using a library of Kme3 analogues (Figure 14). Here, it was observed that KDM4 prefers to associate to Kme3 in an anti-conformation, which was confirmed by an analogue substrate containing a conformationally rigid 4,5-trans double bond that was demethylated with similar efficiency to natural substrate [117]. However, the same result was not obtained for KDM7B, which suggests Lys association in a “syn-type” conformation, indicating the importance of side chain flexibility and potential selectivity towards conformationally restricted substrates [117]. Loss of charge through incorporation of carba- or methoxy-containing analogues led to a complete loss of catalytic demethylation activity mediated by KDM4, highlighting the importance of the positively charged trimethylammonium group for efficient catalysis by KDM4. However, it is important to note that O-demethylation by other 2OG oxygenases has been observed during morphine biosynthesis, indicating substrate specificity towards charged substrates for KDM4s [118]. Taken together, these observations can lead towards full characterization of the mechanism underlying JHDM-catalyzed demethylation and inhibitor studies [117]. As for writing enzymes, trimethylthialysine was also evaluated as an easily accessible mimic for trimethyllysine in the context of erasers. It was shown that trimethylthialysine was accepted as a poor substrate for Jumonji histone demethylase JMJD2A [119]. Interestingly, trimethylthialysine displayed a fourfold slower turnover rate but a fivefold tighter binding with the catalytic pocket of the enzyme.

## 5. Reading Kme3

Methylation of lysine results in chemically distinct ligands, which are recognized by diverse classes of reader domains that initiate cellular processes [9,16,120]. The size of lysine’s side chain increases with each additional methylation, while maintaining the overall +1 charge of the ε-amine at physiological pH. This charge can be dispersed over the surrounding methyl groups in higher methylation states. Additionally, each methylation decreases lysine’s ability to act as a hydrogen bond donor and acceptor, making Kme3 unable to participate in hydrogen bonding [8,11,121].

Kme3 unique characteristics can be recognized by reader protein domains that exhibit a conserved aromatic cage with up to four aromatic residues [16]. Readers of Kme3 include chromodomain, Tudor domain, Pro-Trp-Trp-Pro (PWWP) domain, plant homeodomain (PHD) zinc fingers, and ADD (ATRX-DNMT3-DNMT3L) domain proteins [122]. These domains typically display specificity for the higher methylation states (Kme3 > Kme2 > Kme >> K) [123] through Van der Waals and cation–π interactions, with the methylated ε-amino group due to the increased ability of Kme3 to participate in cation–π interactions caused by the altered charge dispersion. This coulombic attraction is preferred in aqueous solutions over forming a salt bridge with a carboxylate group, as a result of high desolvation free energy penalty [124]. Additionally, a decreased desolvation energy penalty upon binding of Kme3 to the reader domain results in enzymatically favorable release of high energy water molecules that occupy the aromatic cage in its unbound state [11]. The size and amino acid composition of the aromatic cage further dictate specificity for the methylation state of lysine. Reader domains with small aromatic cages sterically prevent Kme3 from binding due to its increased size. Some reader domains contain half-cages composed of aromatic residues and a negatively charged residue that mediates favorable hydrogen bonding with Kme or Kme2 residues [125].

### 5.1. Kme3 Reader Domains

#### 5.1.1. Recognition of Kme3 by Readers of the Royal Superfamily

Protein domains of the Royal Family proteins (chromodomain, chromobarrel, PWWP, Tudor domain) are composed of structurally conserved Src homology 3-like β-barrel topologies that recognize Kme3-containing ligands through an aromatic cage that mediates cation–π interactions [122]. Recognition of ligands by members of this family is often associated with chromatin condensation, transcription, silencing, repair, and maintenance of posttranslational modifications [120].

**Figure 15:**
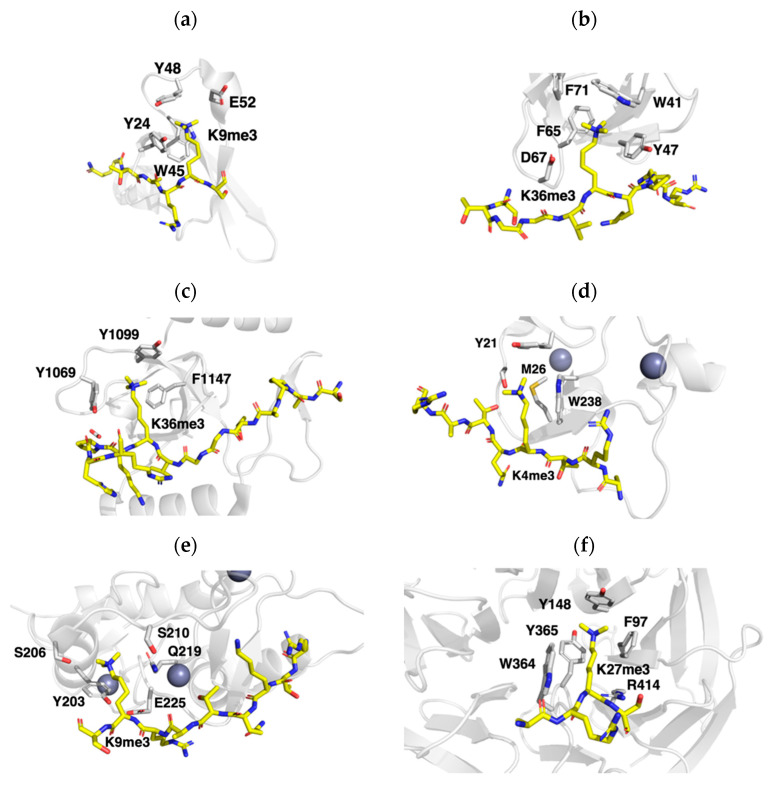
Ribbon representations of Kme3 readers (grey) in complex with histone 3 peptides (yellow) in stick representation. Key residues for Kme3 recognition are labeled and zinc ions are depicted as spheres. (**a**) HP1_CD_ (PDB-ID: 1KNE) [138], (**b**) PHF1_TD_ (PDB-ID: 4HCZ) [139], (**c**) BRPF1_PWWP_ (PDB-ID: 2X4W) [140], (**d**) ING2_PHD_ (PDB-ID: 2G6Q) [141], (**e**) ATRX_ADD_ (PDB-ID: 3QL9) [142], (**f**) EED_WD40_ (PDB-ID: 3JZG) [143].

The evolutionary related chromatin organization modifier (Chromo) domain, double chromo domains, and chromobarrel domain were the first classes of reader domains that displayed specific binding towards methylated lysine (Figure 15a). These classes are made up out of four curved β-strands and an α-helix, which mediate histone peptide recruitment in a β-strand conformation through hydrogen bonding and electrostatic interaction between two chromodomain β-strands. Induced-fit binding subsequently allows for correct positioning of the methylated lysine within the aromatic cage for read-out [126], while further increasing sequence specificity through aromatic pockets that target neighboring Ala residues. Most chromodomains have a preference for binding Kme3 in both H3 and H4 [126,127,128]. For example, HP1 and structurally similar polycomb chromodomains displayed binding of H3K9me3 and H3K27me3, respectively. However, mouse or *Drosophila* HP1 aromatic cages are contain Asp or Glu residues, which allow for H3K9me2 binding [129].

Tudor domains are characterized by a singular or tandem five-stranded β-barrel domain that mediates the recruitment of the histone tail to the groove within the barrel. Recruitment of the histone tail leads to insertion of Kme2/3 or methylated arginine residues into an aromatic cage (Figure 15b) [130,131]. Tudor domains can be distinguished as three forms: single Tudor, tandem Tudor, and hybrid Tudor domains. They have been characterized to target H4K20me3 (JMJD2A, 53BP1), H3K36me3 (JMJD2A, Sgf29, Spindlin1), and H3K4me3 (PHF1, PHF19) [131]. Similar to chromo domains, the aromatic cage is flanked by binding pockets that further define ligand sequence specificity [132]. The majority of the tandem Tudor domains bind the histone tail using only one β-barrel. However, H3K4me3 binding by Sgf29 is characterized by binding of H3A1 and H3K4me3 by separate β-barrels [133]. Another exception is the three Tudor domains of Spindlin1, which exhibits two binding modes for H3 ligand binding. It can recognize H3K4me3 or can simultaneously interact with H3K4me3 and H3R8Me2a through a folded globular domain consisting out of a tandem Tudor domain followed by a single Tudor domain. H3K4me3 is normally bound in an extended conformation that specifically interacts with the second Tudor domain. However, methylation of R8 leads to a reorientation of the Arg side chain towards the first Tudor domain for high affinity interactions [120,134].

Pro-Trp-Trp-Pro (PWWP) domain-containing proteins are associated with comparatively weak binding of H3K36me3 and H4K20me3, which are marks of transcriptional repression (Figure 15c). In general, the PWWP domains can be divided into three units: β-barrel, insertion motif, and a C-terminal α-helical bundle. The Kme3-binding aromatic cage is semi-conserved and assumes a similar fold to chromodomains [135]. In addition, the domain can recruit DNA through electrostatic interactions with the phosphate backbone [136]. BRPF1, for example, can bind to H3K36Me3 through various interactions between a surface groove between the β-barrel and α-helical insertion and the ligand. In addition, PWWP domains exhibit conserved hydrophobic pockets that interact with H3T32 and H3Val35 to further increase binding affinity. The exact biological relevance of PWWP domains is still undetermined [137].

#### 5.1.2. Recognition of Kme3 by PHD Zinc Fingers

The family of plant homeodomain (PHD) zinc fingers have been characterized to bind various states of lysine methylation (Figure 15d) [144,145,146]. Their globular fold is comprised out of a β-sheet and an α-helix, which is dependent on a Cys_4-_His-Cys_3_ motif that chelates two zinc ions in a cross-brace manner. Like readers of the Royal Family, the PHD domains contain an aromatic cage for Kme binding. Depending on the subtype of PHD finger, the histone tail is bound in an extended, bent, or helical conformation to insert Kme3 into the aromatic cage. PHD fingers that target H3K4me3 complex to the histone tail in a β-strand conformation form a third antiparallel β-strand that allows for the insertion of the methylated lysine into the aromatic cage for read-out [144,145,146]. The sequence surrounding the aromatic cage dictates sequence specificity. For example, the PHD finger of ING2 complexes the H3K4me3 tail in an extended conformation that inserts the N-terminal Ala1 into a hydrophobic pocket. The terminal amine can provide hydrogen bonding interactions with the carbonyl backbone of the domain. Ionic and hydrogen bonding interactions with Arg2 are maintained by a separate pocket with acidic residues [120,141].

The ADD (ATRX-DNMT3-DNMT3L) domain consists of a non-canonical PHD finger and a GATA-like zinc knuckle. The ADD domain of ATRX specifically interacts with H3K9me3 in a combinatorial manner where the DNMT3 and DNMT3L domains recognize H3K4 (Figure 15e). The GATA-like zinc knuckle within the ATRX_ADD_ binds H3K9me3 in a non-aromatic pocket, resulting in a unique read-out where the trimethylammonium group is coordinated with a singular aromatic residue Y203 and non-conventional carbon-oxygen hydrogen bonds [142,147,148].

#### 5.1.3. Recognition of Kme3 by WD40 Zinc Fingers

WD40 beta-propeller domain-containing proteins (Figure 15f) are often found in chromatin-associated multi-protein complexes. The WD40 fold is associated with promiscuous read-out activities, as it was observed to interact with Kme0/3 or Rme2 on various histone proteins through an aromatic cage. Four repeated β-propeller units provide multiple docking sites for the recruitment of chromatin remodeling proteins. However, the exact number of repeats and the sequence can vary between family members. Structural analysis of EED_WD40_ revealed recognition of histone tail through interactions with the aromatic cavity on the surface of the beta propeller of the reader domain in a solvent exposed manner. EED reads repressive chromatin marks H3K27me3, H3K9me3, H4K20me3, and H1K26me3 with an aromatic cage. Specificity for these marks is mediated by a flanking hydrophobic, a solvent-exposed, and another a flanking hydrophobic residue at positions −2, 1, and +2 relative to the Kme, respectively. These cavities can only accommodate the smaller residues that are associated with the repressive histone peptides, driving selectivity [143,149].

### 5.2. Modification of Reader Proteins to Investigate Biomolecular Recognition

Subtle modifications to the residues in the aromatic cage of Kme3 readers were made to investigate the strength of the cation–π interactions during readout of the Kme3-containing histones. Mpp8 chromodomain recognizes H3K9me3, and its binding affinity was negatively affected by incorporation of electron-deficient fluorinated phenylalanines containing two to five fluorine substituents within the aromatic cage on position 59 [150]. An increased number of fluorine substituents decreased the H3K9me3 binding affinity further due to loss of crucial cation–π interactions, which are being disturbed by fluorine’s electronegative properties. In a physic-organic study, fluorinated tryptophan residues were introduced into the aromatic cage of the KDM5A PHD3 zinc finger. It was found here that the fluorinated aromatic cages generally associated with Kme3 with the same binding affinity as did nonfluorinated cages; however, it was observed that the overall cation–π interactions were weaker in the fluorinated cages, yet these were compensated by more favorable release of high-energy water molecules in a Kme3-mediated process [151].

The residues within HP1 chromodomain’s aromatic cage were substituted for more electron-rich analogues, which resulted in increased binding affinity for H3K9me3 [150]. In contrast, electron-poor tyrosine analogues led to loss of binding affinity, further indicating the importance of cation–π interactions in strong binding of Kme3 ligands [150]. HP1′s aromatic cage residues were individually substituted on position 24 and 48 with tyrosine analogues to further establish the individual contributions of these two residues in recognition of Kme3, revealing that the residues do not equally contribute towards cation–π binding and that efficient association depends on contact between Tyr residues and Kme3 [152]. Additionally, the Tyr residues present in the HP1 aromatic cage were substituted by Trp to investigate if this mutation could increase the binding strength to the Kme3-containing substrate, and more generally the conservation of aromatic residues within aromatic cages [153]. It was found that the mutation Y24W was not perturbing and allowed in the binding pocket, leading to a −5 kcal mol^−1^ stronger binding.

Mutational analysis of half-cages containing negatively charged residues were indicative of the role that electrostatic interactions play in the readout and selectivity towards of Kme2/Kme3-containing ligands. HP1E52 was replaced by neutral amino acids in an attempt to increase selectivity towards H3K9me3 by weakening hydrogen bonding and electrostatic interactions necessary for efficient H3K9me2 binding while maintaining affinity for H3K9me3 [129]. This modification led to increased specificity towards H3K9me3, and 3.5-fold weaker binding to H3K9me2, emphasizing the importance of electrostatic interactions and hydrogen bonding in mediating selectivity towards Kme2 readout. Similar results were obtained in studies that modified D266 through site-directed mutagenesis in SGF29′s Tudor domain while also demonstrating the importance the negative charge in providing structural integrity of the reader domain complex [130].

### 5.3. Recognition of Kme3 Analogues by Epigenetic Readers

**Figure 16:**
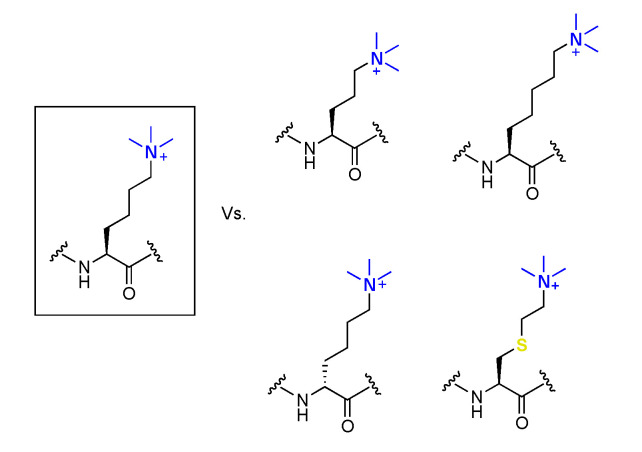
Kme3 analogues recognized by epigenetic reader domain proteins.

**Figure 17:**
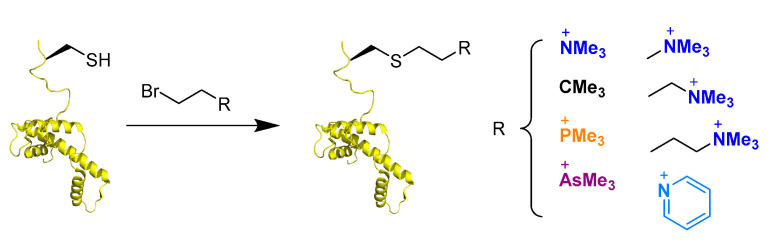
Analogues of Kme3 generated by Cys4 alkylation of histone H3 and recognized by the PHD3 reader domain of JARID1A. (Reprinted (adapted) with permission from *Bioconjugate Chem.* 2019, 30, 3, 952–958. Copyright 2020 ACS Publications.).

The importance of Kme3 side chain’s length and stereochemistry was explored towards mediating Kme3 readout (Figure 16) [68,154]. Increasing or decreasing the aliphatic chain length with one carbon by incorporation of trimethylornithine or trimethylhomolysine into histone peptides led to a slight decrease in binding affinity and enthalpy of binding for readers of the histone code compared to WT peptides [154]. Kme3 thus has an optimal chain length for positioning Kme3 into the aromatic cage, eliciting stronger cation–π interactions. A panel of readers of Kme3 (KDM5A_PHD3_, TAF3_PHD_, BPTF_PHD_, SGF29_PHD_, and KDM4A_TTD_) were experimentally and computationally shown to recognize histone peptides containing D-Kme3 stereochemistry with an 8–36 time decrease in affinity compared to L-Kme3-containing histones (Figure 16). A less favorable enthalpy was observed, possibly due to the inherent flexibility of the side chain that reorients D-Kme3 in the aromatic cage to mediate favorable, but still weaker cation–π interactions [68]. Additionally, Kme3 readers can recognize histone peptides with cysteine-derived K_c_me3 with affinities comparable to Kme3. The analogue replaces a side chain hydrocarbon with sulfur and has slightly altered properties compared to lysine (Figure 16) [155,156,157]. These results put forward the idea to use cysteine-derived K_c_me3 as Kme3 mimics in studies using intact histones or a full nucleosome, as C110 on H3 is the only naturally occurring cysteine residue in all of the histone proteins [142]. This allows for site-specific incorporation of Kme3 analogues through a straightforward and selective alkylation of cysteine residues that were incorporated into the histone sequence via point mutagenesis [156,157]. HP1 was shown to recognize full-length H3K_c_9me3 in the context of the nucleosome assembly [157,158,159]. Surprisingly, two separate studies reported that in one case, trimethylthialysine was well accepted by reader proteins [156], while the other report states that trimethylthialysine is in fact not a good mimic of Kme3 [155]. Recently, a new physical-organic study on another set of reader proteins, namely, KDM5_PHD3_, TAF3_PHD_, BPTF_PHD_, SGF29_TTD_, and KDM4A_TTD_, showed that binding of these domains was practically indistinguishable between Kme3- and K_c_me3-containing peptides, indicating that the thia derivative of Kme3 indeed can be used in biomolecular studies [160]. Having established this, the straightforward alkylation chemistry can still be employed to easily generate analogues of Kme3. To this end, a panel of cysteine-derived analogues of Kme3 was incorporated on full-length H3 at position 4; such histones were recognized by the PHD3 reader domain of human JARID1A (Figure 17) [161]. These analogues could also be incorporated into histone octamers effectively.

## 6. The Role of Kme3 on the Nucleosome Assembly

Lysine methylation can exert indirect regulation of gene expression through recruitment of effector proteins with reader domains of the diverse lysine methylation states, while also controlling the chromatin and nucleosomal structure directly [157,162]. Trimethylated lysine residues have been found on several sites on histone proteins—H3K4me3 and H3K36me3 are linked with gene activation, whereas H3K9me3, H3K27me3 and H4K20me3 with gene repression [163]. Electrostatic interactions between the histone octamers and/or the DNA are in part mediated by the charged lysine residues found in the histone tails or histone core surface [164]. Acetylation of H4K16 results in neutralization of the positive histone protein charge, weakening the electrostatic interactions between the histone protein and DNA, which results in decreased chromatin condensation, thus leading to activation of transcription [165]. The various lysine methylation states were similarly predicted to affect the macromolecular interactions within the nucleosome [157]. However, unlike lysine acetylation, lysine methylation does not drastically alter the electrostatic properties of the residue (Figure 1b) [8,9,10]. Instead, trimethylation of lysine results in a slight increase in side chain bulk, which mediates conformational change within the histone tail, with H4K20me3 displaying increased chromatin condensation. New chemical strategies were developed to investigate the exact role of lysine methylation patterns of any histone protein in nucleosome assembly and chromatin compaction [162]. These methodologies span native chemical ligation (NCL) or expressed protein ligation (EPL), installation of PTM mimics, or genetic methyllysine installation [125,157,162,166,167,168].

NCL and its extended method EPL can provide a valuable semi-synthesis-based approach for studying the effects of lysine methylation in histones, combining the strengths of expression- and synthesis-based methods [169]. Unlike linear peptide synthesis, two or several linear peptides are ligated together, which allows for the synthesis of entire proteins [170,171]. This technique becomes especially valuable when studying unnatural or post-translationally modified proteins, as these are often difficult to obtain using expression-based methods. NCL allows for complete freedom of the choice of amino acid chain, alleviating the difficulty of introducing post-translationally modified peptides. Furthermore, native chemical ligation allows for the site-specific introduction of these kind of amino acids, so that single PTMs or their combination can be studied effectively [164]. With expressed ligation, a modified histone peptide containing a carboxy terminal thioester can be synthesized with high levels of synthetic control and subsequently ligated to a recombinant histone core protein with a mutated terminal cysteine residue. Exemplifying this approach, acetylated and methylated histone H3 and H4 proteins were generated semi-synthetically [168]. This allowed for the introduction of selective, naturally occurring PTMs into histone proteins, and subsequent formation of histone octamers and nucleosome arrays, which may lead to the development of a better understanding of the epigenetic signaling mechanisms. As not all PTMs on histones occur on the N-terminal tails, but also in the core region, a fully synthetic approach would be needed to study the effect of these modifications. To this end, a three-fragment strategy was developed to generate the fully synthetic H3K9me3 and H3K4me3 proteins [172,173]. Although (semi)synthetic approaches enable preparation of histone proteins containing Kme3, such strategies are time-consuming, technically challenging, and produce small amounts of proteins of interest.

The synthetic or semi-synthetic histones can be incorporated into nucleosomes to allow for detailed studying of the effect of PTMs on nucleosomal compaction. Semi-synthetic H3K4me2 and H3K4me3 were incorporated on the nucleosomal level and investigated in terms of how the PHD domain of BPTF interacts with these marks, in conjunction with the neighboring bromodomain that recognize H4K16ac [174]. It was found that there exists an interplay between the two separate histones, influencing the binding of BPTF in a synergistic way, suggesting that crosstalk between PTMs on different histones is an important mechanism of regulation in the nucleosomal context.

**Figure 18:**
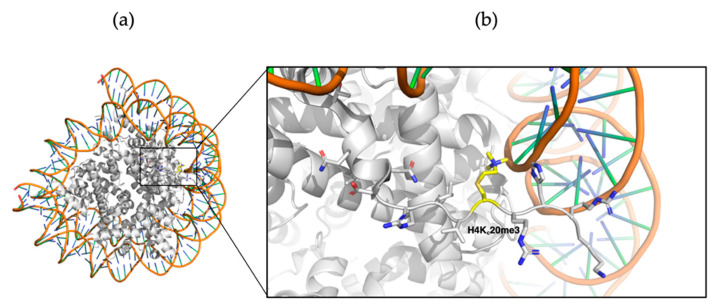
(**a**) Ribbon representations of the nucleosome assembly, showing the histone octamer (grey) in complex with DNA (orange). The K_c_me3 residue on histone 4 is shown in yellow (PDB-ID: 3C1B). (**b**) A zoomed view on H4K_c_20me3 pointing towards the DNA.

Cysteine-derived K_c_me3 was shown to closely mimic Kme3 residues, and was generally accepted for read-out by epigenetic proteins [76,119,155,156,157]. The low occurrence of cysteine (one or two Cys residues are found on all four nucleosomal histones) within the histone tail and the reactive properties of the cysteine side chain nucleophile makes the residue a prime candidate for site-specific methylation through alkylation [142]. Expression of modified cysteine-containing histone protein (H3C4, H3C9, H3C36, H3C79, and H4C20), subsequent alkylation to K_c_/K_c_me/K_c_me2/K_c_me3, followed by nucleosome assembly was demonstrated to be rapid and high-yielding, while not disturbing nucleosome accessibility for epigenetic proteins [157,162]. In a follow-up study, crystal structures were generated with the site-specifically modified nucleosomes with H3K_c_79me2 and H4K_c_20me3 to investigate the role of lysine methylation on the structure of the nucleosome assembly. Neither modification was shown to negatively influence nucleosome assembly and it was shown that H4K_c_20me3-containing nucleosomes required less Mg^2+^ to mediate chromatin compaction compared to unmethylated H4K20. H4K_c_20me3 affects the orientation of surrounding residues to increase chromatin compaction, with His18 now forming a hydrogen bond with DNA, while orientating the methylated side chain to the DNA backbone (Figure 18) [162]. Compared to NCL, no native cysteine residue is required within the sequence of interest to mediate the ligation, making it less laborious.

Genetic methyllysine installation with bacterial systems has allowed for site-specific incorporation of Kme and Kac. Orthogonal pyrrolyssyl-tRNA synthethase and tRNA_CUA_ allow for insertion of these modified lysine residues in response to the amber codon [166]. To incorporate Kme, N^ɛ^-tert-butyloxycarbonyl-N^ɛ^-methyl-L-lysine was installed in the protein and subsequently deprotected using mild conditions. A combination of genetic code expansion and chemoselective chemistry has allowed for site-selective Kme2 installation with low yields [167]. This was achieved by incorporating a protected lysine residue to an amber codon, after which all other amino-containing residues were protected under mild conditions. Subsequent selective deprotection of the target lysine and reductive methylation yield Kme2, which can be followed by a final deprotection to yield the modified full-length protein H3K9me2 that was successfully synthesized using this method, and displayed specific binding to HP1 [166,167]. To date, there has been no report using the same strategy for incorporation Kme3 into proteins.

## 7. Summary and Perspectives

In this review, we discussed the role of Kme3 as an intermediate for the carnitine biosynthesis pathway, the enzymatic processes involved in generation and removal of Kme3, Kme3 recognition by epigenetic reader domains, and the role of Kme3 on the structure and function of the nucleosome. We also described experimental work that has been done to elucidate the underlying biocatalytic and binding mechanisms that are involved in generation, removal, and recognition of Kme3; the role of Kme3-hydroxylation in carnitine biosynthesis; and the synthesis of full-length histone protein possessing Kme3 and its simplest analogues to study the nucleosome assembly and chromatin structure.

A better molecular knowledge of the carnitine biosynthesis pathway in human needs a more profound mechanistic understanding of the four enzymes involved, as well as development of highly active and selective chemical probes for in vitro and in vivo studies. To date, only BBOX has been targeted by small molecules [175], in particular by Meldonium, a clinically used anti-ischemic drug [176]. Inhibitors of TMLH, however, have not been developed yet, but would be of relevance for potential therapeutic intervention for cardiovascular diseases.

Current knowledge on PTM read-out by reader domains and desire to further analyze, understand, and quantify lysine methylation has led to the development of synthetic receptors through host–guest chemistry that specifically recognizes methylated lysine residues via cation–π interactions [177,178,179]. Tools such as these might form the basis for epigenome profile read-out and play a role in the development of personalized medicine schemes [180]. Various epigenetic drugs and diagnostic biomarkers have entered or passed clinical trials. Development of inhibitors targeting epigenetic enzymes has mainly targeted histone deacetylases and DNA/histone methyltransferases, resulting in emergence of new anti-cancer therapies [181,182]. However, highly dynamic and context-sensitive crosstalk between PTMs, downstream signaling effects mediated by PTMs, and the discovery of new modifications introduce more levels of complexity and illustrate the challenges that need to be overcome to fully decipher the epigenetic code. With epigenetic dysregulation playing a key role in health and disease, it is now more important than ever to develop chemical tools and inhibitors to investigate methylation of lysine, among other PTMs, in (non-)histone proteins to shed more light on the dynamic and complex nature of PTMs. Exploration of the epigenetic chemical space through chemical biology approaches will pave the way towards a more complete understanding of the underlying molecular epigenetic mechanisms.
